# High-density lipoprotein of patients with Type 2 Diabetes Mellitus upregulates cyclooxgenase-2 expression and prostacyclin I-2 release in endothelial cells: relationship with HDL-associated sphingosine-1-phosphate

**DOI:** 10.1186/1475-2840-12-27

**Published:** 2013-01-30

**Authors:** Xunliang Tong, Hui Peng, Donghui Liu, Liang Ji, Chenguang Niu, Jun Ren, Bing Pan, Jianying Hu, Lemin Zheng, Yining Huang

**Affiliations:** 1 Department of Neurology, Peking University First Hospital, Beijing 100034, China; 2 The Institute of Cardiovascular Sciences and Institute of Systems Biomedicine, School of Basic Medical Sciences, and Key Laboratory of Molecular Cardiovascular Sciences, Ministry of Education, Key Laboratory of Cardiovascular Molecular Biology and Regulatory Peptides, Ministry of Health, Peking University Health Science Center, Beijing 100191, China; 3 College of Urban and Environmental Sciences, Peking University, Beijing 100871, China; 4 Division of Kinesiology and Health & Biomedical Science PhD Program, University of Wyoming College of Health Sciences, Laramie, WY, USA

**Keywords:** Type 2 diabetes mellitus, High-density lipoprotein, Sphingosine-1-phosphate, Cyclooxygenase-2, Prostacyclin I-2, Endothelial cells

## Abstract

**Background:**

Dysfunctional high-density lipoprotein (HDL) may have pro-inflammatory effects on the endothelial cells,which causes atherosclerosis in type 2 diabetes mellitus (T2DM). HDL is a major carrier of sphingosine-1-phosphate (S1P) in plasma while S1P exhibits multiple biological activities. However, potential role of HDL and S1P in T2DM remains unexplored. We hypothesized that diabetic HDL with higher contents of S1P exerts beneficial effects on the vascular system.

**Methods:**

Subjects with T2DM with or without proved large arteries atherosclerosis and normal controls (n=15 for each group) were recruited in the present study. HDL was isolated from the subjects by ultracentrifugation. The levels of HDL-associated S1P were determined by UPLC-MS/MS. The protective function of diabetic HDL and S1P was evaluated by measuring cyclooxygenase-2 (COX-2) expression and prostacyclin I-2 (PGI-2) release by human umbilical vein endothelial cells (HUVECs) using western blot and enzyme-linked immunosorbent assay (ELISA), respectively.

**Results:**

The S1P levels in isolated HDL were significantly increased in T2DM subjects compared with controls (235.6 ± 13.4 vs 195.0 ± 6.4 ng/mg, P< 0.05). The diabetic HDL exerted greater protective effects on inducing COX-2 expression and PGI-2 release by HUVECs than those of control HDL (p < 0.05, p < 0.01, respectively). Pertussis toxin, a common inhibitor of G-couple protein receptors, and VPC 23019, an antagonist of S1P receptor 1 and 3 significantly attenuated HDL-induced COX-2 expression and PGI-2 release.

**Conclusions:**

Diabetic HDL carries higher level of S1P compared with normal HDL, which has the potential to contribute to protective effects on endothelial cells by inducing COX-2 expression and PGI-2 release. These findings provide a new insight of S1P function in T2DM patients, possibly leading to a new therapeutic target.

## Introduction

The rising incidence and complications of type 2 diabetes mellitus (T2DM) are becoming major public health problems [1]. One of the most serious complications of T2DM is atherosclerosis, a vascular disease, which accounts for 80% of diabetic mortality. Due to the close links between atherosclerosis, T2DM and cardiovascular diseases, endothelial self-protection and dysfunction are more considered in therapeutic strategies of T2DM [2]. High-density lipoprotein (HDL) conveys an impressive spectrum of vascular-protective properties. In addition to its role in reverse cholesterol transport, HDL also exerts beneficial effects on anti-oxidative, anti-inflammatory, anti-thrombotic and endothelium-dependent vaso-relaxation [3]. It has been shown that the impaired HDL and apolipoprotein A-I (apoA-I) function contributes, at least in part, to the development of T2DM-mediated atherosclerotic vascular disease[4-6].The growing evidence indicates that sphingosine-1-phosphate (S1P) and HDL exhibit their biological activities, either alone or in a combined version [7-9].

HDL normally services as a plasma carrier of S1P and provides a platform for S1P activities. S1P is generated from sphingomyelin, one major lipid in the lipid bilayer of the cell membrane. It is a lipid component of HDL. S1P is a mediator of many of the cardiovascular protective effects of HDL, including the ability to inhibit/reverse atherosclerosis [9,10]. Atherosclerosis is a multi-factorial inflammatory disease in which cyclooxygenase (COX) and the downstream diverse prostanoids may play important role in the development of the disease [11]. It is now known that the two genes encored two similar but distinct isoforms of the enzyme, COX-1 and COX-2 [12]. COX-1 is a non-reducible form of COX protein while the expression of COX-2 can be regulated [13]. S1P associates with HDL exerting synergic function in induction of the COX-2 expression and prostaglandin I-2 release [10,14,15].

Although previous studies have shown that in patients with T2DM the protective functions of HDLs are impaired [16], it still remains uncertain if these phenomena differ from various stages of the disease, particularly in the beginning of it. We hypothesized that the HDL with increased content of S1P plays a compensatory-protection role in vascular system in T2DM.

## Materials and methods

### Subjects

The study was approved by the Institutional Review Board and the Ethic Committee of Peking University First Hospital, Beijing, China, and subjects provided written, informed consent. The inclusion criteria of T2DM group were 40–75 years old subjects with T2DM diagnoses in accordance with international standards, fasting plasma glucose (FPG) ≥ 7.0 mmol/L and glycated hemoglobin (HbA1c) > 6.5%, and T2MD was first diagnosed without any treatment before. Exclusion criteria of T2DM group were a history of macrovascular (cardiovascular and cerebrovascular events) and microvascular disease (nephropathy absence of albuminuria, retinopathy and peripheral neuropathy), usage of insulin and statin treatment before. The inclusion criteria of T2DM accompanied with atherosclerosis (T2DM-As) group were 40–75 years old subjects with T2DM diagnosed in accordance with international standards, fasting plasma glucose (FPG) ≥ 7.0 mmol/L and glycated hemoglobin (HbA1c) > 6.5% and confirmed history of macrovascular atherosclerosis (cardiovascular and cerebrovascular) verified by ultrasound, computerized tomography angiography (CTA) or magnetic resonance angiography (MRA).

### Cell culture and western blotting

Human umbilical vein endothelial cells (HUVECs) were isolated from freshly obtained human umbilical cords by collagenase digestion of the interior of the umbilical vein as described previously [17]. Cells were used for experiments prior to their forth passage. Briefly, cells were cultured in gelatin-coated polystyrene dishes and propagated in endothelial cell medium (ECM) (ScienCell, CA) supplemented with 5% FBS and endothelial cell growth supplement (ECGs) (ScienCell, CA) at 37°C in a 5% CO2-humidified atmosphere. Western blotting was performed as previously described [18]. After incubation with or without stimuli, the cultured HUVECs were washed with ice PBS for three times and then lysed in radioimmuno precipitation assay buffer. The protein concentration was measured with bicinchoninic acid (BCA) method according to the instructions of the company (BCA kit, Pierce, Rockford, IL). An equal amount of the total protein was subjected to electrophoresis and then transferred to nitrocellulose membranes, incubated in blocking buffer (1% bovine serum albumin in Tris-buffered saline with 0.1% Tween-20) for 2 hours. The COX-2 protein was detected by incubating with antibody against human COX-2 (Cayman Chemical, USA) for eight hours. After washing, the membrane was incubated in blocking buffer for 90 min at room temperature and probed with either HRP-goat-anti-rabbit IgG or HRP-goat-anti-mouse IgG (MBL, Japan) secondary antibodies subsequently developed with Western blotting detection regents by ECL (Piece Biotechnology, IL) according to the manufacture’s instruction. The densities of specific immunoreactive protein bands were quantified using Quantity One 1-D Analysis Software (Bio-rad, Hercules, CA).

### Isolation of HDL

Fasting venous blood samples were drawn on EDTA tubes from subjects after a 12 h fasting food. Plasma was immediately collected after centrifugation at 4°C 3,000 rpm for 10 min. HDL were then isolated from fresh EDTA plasma by the method of density gradient ultracentrifugation (d=1.13–1.21 g∕ mL) as previously described [19]. The isolated HDL was further purified in dialysis of PBS, sterilized through a 0.22 um filter and stored at 4°C for the further usage within a month of isolation. The purity of the HDL was confirmed by SDS-PAGE and western blot using goat anti-apoA-I polyclonal antibody (DiaSorin, Stillwater, OK), then quantified through the measurement of apoA-I content by nephelometry (Dimension XPand, Dade Behring, Germany). HDL2 (1.063<d<1.125 g/ml) and HDL3 (1.125<d<1.21 g/ml) were isolated by sequential ultracentrifugation as previously described [20]. HDL subfractions were dialyzed against saline/EDTA (150 mM NaCl, 300 μM EDTA, pH 7.4), sterilized by filtering through a 0.22 μm membrane, and stored at 4°C until used. Equal concentration of apoA-1 in isolated HDL was used for cell treatment and S1P level determination.

### Detection of levels of HDL-associated S1P

UPLC-MS/MS technique was employed to measure levels of HDL-associated S1P as previously described [21]. Briefly, after loading of HDL samples (200 μL for each test) to a 1.5 ml centrifuge tube, methanol (800 μL) and methanol with standard C17-D-erythro-sphingosine-1-phosphate (C17-sph) (50 μl of 1000 μg/L) were added to the same tube and then shaken vigorously for 1 minute. After centrifugation at 12,000 rpm for 15 minutes, the supernatant was collected and analysis of S1P was performed by a Waters ACQUITY UPLCTM system. Waters ACUITY UPLC BEH Phenyl column (1.8 μm; 2.1 mm × 100 mm) was selected for chromatographic separation. The injection volume was 5 μL. Methanol (A) and 0.5% formic acid in ultrapure water (B) were used as mobile phases. The gradient started at 10% A and then increased linearly to 60% in 6 minutes, to 100% at 6 minutes and kept for 2 minutes, followed by a decrease to initial conditions of 10% A and held for 2 minutes to allow for equilibration. The flow rate was 0.3 mL/min. The column was maintained at 40°C, and the sample room temperature was 10°C.

S1P was analyzed by MS/MS in the multiple-reaction monitoring (MRM) mode. The optimized MS/MS parameters for the analyses including their precursor and product ions, cone voltage, and collision energy were shown in Table 1.

**Table 1:** Optimized instrumental and MRM conditions of S1P and C17-sph

**Compound**	**Dwell time (s)**	**Precursor ion**	**Cone voltage (V)**	**Product ion**	**Collision energy (eV)**
S1P	0.1	380	25	265	18
				82	40
C17-sph	0.1	366	25	251	18
				82	40

Eight points calibration curves for S1P were constructed for the standard solutions between 7.8 and 1000 μg/L, and calibration graphs were linear with good correlation coefficients (r^2^>0.99). Recoveries of target compounds were 89±4% in isolated HDL by spiking standards of S1P (500 ng/mL) to HDL samples (n=3). The limits of detection (LODs) were estimated to be 30 ng/mL for HDL based on the peak-to-peak noise of the baseline and on a minimal value of signal-to-noise of 3 and LOD, respectively. Concentrations of S1P in plasma or HDL samples were corrected by C17-sph.

### ELISA for quantitation of 6-keto PGF1α production

HUVECs were cultured with or without various HDL or other reagents for 6 hours. After washing with phosphate buffered saline (PBS) at 37°C, cells were incubated with 1 mL of arachidonic acid (10 μmol/L in 0.75% fatty acid-free BSA/PBS buffer) at 37°C for further 30 minutes. Supernatants were collected for measurement of production of 6-keto PGF1α, an inactive, non-enzymatic hydrolysis product of PGI-2. Concentrations of 6-keto PGF1α in the supernatants were determined using a 6-keto PGF1α EIA kit according to the manufacturer’s instructions (Cayman Chemical, USA). Limit of detection was 6 pg/mL.

### S1P associated with BSA and N-HDL

Fatty acid-free BSA and N-HDL were used as a carrier for exogenous S1P. S1P (2 nM) was added into tubes containing 30 μg/ml of BSA or N-HDL separately overnight at 4°C for further usage.

### Statistical analysis

The results are presented as the means ± SEM. Data was analyzed using Mann–Whitney U test and Student’s *t* test. For all tests, p < 0.05 was considered significant.

## Result

### Study participant characteristics

In total 3 groups (15 healthy controls: 9 female, age 60.53 + 14.46 yrs; 15 patients with T2DM: 8 female, age 56.60 + 14.98 yrs; 15 patients with T2DM-As: 8 female, age 57.40±14.67) participated in the study (Table 2). The subjects with T2DM and with T2DM-As had higher levels of fasting blood sugar, glycated hemoglobin (HbA1C), cholesterol, low density lipoprotein-cholesterol (LDL-c), triglycerides and lower levels of HDL-c than that of the controls (Table 2). T2DM-As group had higher levels of fasting blood sugar, glycated hemoglobin (HbA1C), cholesterol, low density lipoprotein-cholesterol (LDL-c) and triglycerides than that of the T2DM group.

**Table 2:** Subject Characteristics

**Characteristic**	**Healthy controls (n=15)**	**T2DM (n=15)**	**T2DM-As (n=15)**
**Age, years**	60.53±14.46	56.60±14.98	57.40±14.67
**Sex, male/female**	6:9	7:8	7:8
**Fasting glucose (mmol/L)**	5.29±0.35	7.47±2.05^*^	8.47±1.05^*#^
**HbA1c (%)**	5.05±0.35	7.39±0.75^*^	7.73±0.65^*#^
**Triglycerides (mmol/L)**	1.04±0.20	2.13±1.00^*^	2.72±0.59^*#^
**Total cholesterol (mmol/L)**	3.22±0.40	4.70±0.72^*^	5.31±0.33^*#^
**HDL-c (mmol/L)**	1.33±0.11	0.95±0.26^*^	0.93±0.17^*^
**LDL-c (mmol/L)**	1.96±0.23	2.93±0.79^*^	3.25±0.68^*#^

Abbreviations: HDL-c, high-density lipoprotein cholesterol; LDL-c, low-density lipoprotein cholesterol. Values are described as means ± SD.

^*^P<0.001 versus healthy controls.

^#^ P<0.001 versus T2DM.

### Increased level of S1P in diabetic HDL

**Figure 1:**
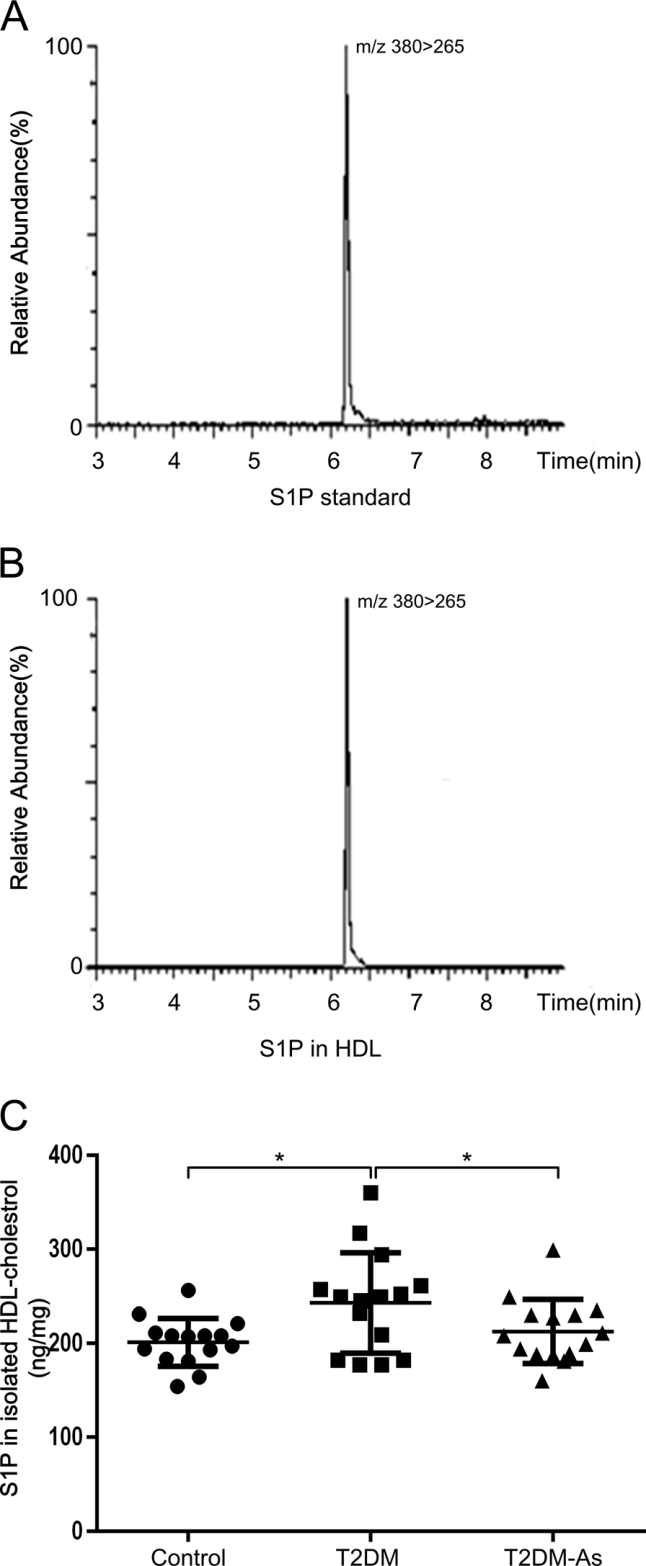
**Concentrations of HDL-associated S1P in diabetes and controls.** Peaks of S1P standard (**A**) and S1P in HDL (**B**) were obtained by UPLC-MS/MS. The S1P in HDL was extracted following the method of methanol precipitation of protein. S1P in isolated HDL-c (**C**) were compared among controls, type 2 diabetes mellitus (T2DM) and type 2 diabetes mellitus accompanied with atherosclersosis (T2DM-As) (n=15 for each group). Mann–Whitney U test. Bars show medians. * p < 0.05.

The peak of S1P standard was detected by the methods described in Method. The HDL-associated S1P was extracted following the method of methanol precipitation of protein, and the S1P level was detected. The figure showed the peak of S1P in standard and isolated HDL (Figure 1A and B). The levels of HDL-associated S1P were significantly increased in T2DM group compared with control group (Figure 1C, T2DM vs controls: 235.6 ± 13.4 vs 195.0 ± 6.4 ng/mg, p< 0.05). The levels of HDL-associated S1P were decreased in T2DM-As group compared with T2DM group (Figure 1C, T2DM-As vs T2DM: 212.5±8.8 vs 235.6 ± 13.4 ng/mg, p< 0.05). There was no statistical difference between T2DM-As and controls.

### Diabetic HDL enhances COX-2 protein expression and induces PGI-2 release

**Figure 2:**
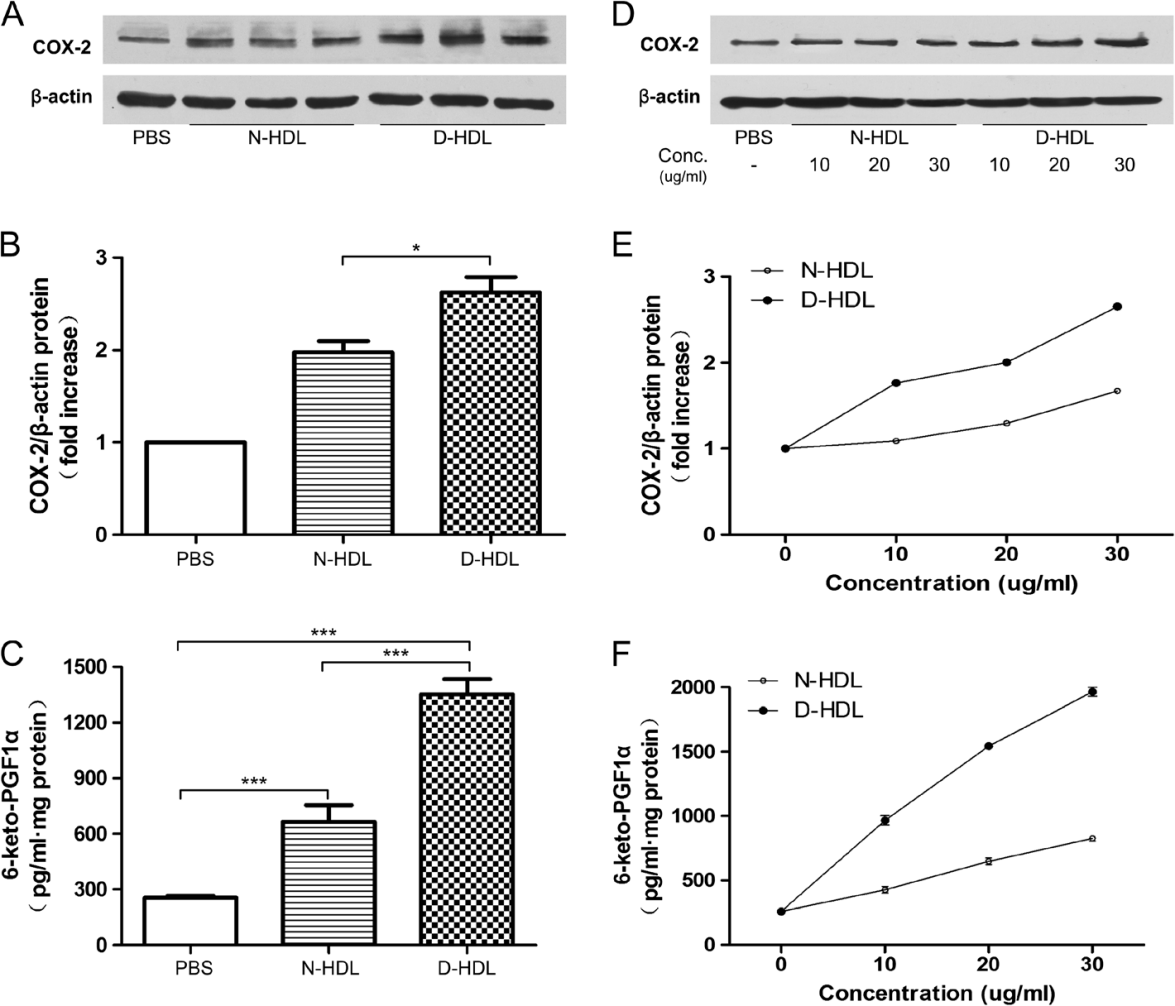
**Diabetic HDL induces HUVEC COX-2 expression and PGI-2 release.** Cells were incubated with N-HDL (HDL isolated from normal controls, 30 μg/mL, n=15), D-HDL (HDL isolated from diabetes, 30 μg/mL, n=15) for 6 hours. Cells were cultured with PBS as a negative control. The expression of COX-2 was assayed by Western blot analysis (**A** and **B**). PGI-2 release was determined by competitive ELISA (**C**). **D**-**F** showing that the effects of HDL on HUVECs were in a concentration-dependent pattern. Data are expressed as the means ± SEM of three independent experiments. Student’s *t* test. * p < 0.05. *** p < 0.001.

Using Western blotting technique, we found that cultured HUVECs spontaneously expressed COX-2 protein at the baseline. This expression was enhanced by HDL of normal controls (N-HDL) and diabetic HDL (D-HDL). Equal concentration of N-HDL and D-HDL (30 μg/mL) was incubated with endothelial cells for 6 hours. It was great interesting of that diabetic HDL significantly up-regulated COX-2 expression by HUVECs than that of HDL from normal controls (Figure 2A and B). Competitive ELISA showed that both sources of HDL significantly induced PGI-2 release (Figure 2C). Again, the effect of diabetic HDL was greater than that of N-HDL (Figure 2C, D-HDL vs. N-HDL: 1351 ± 200.1 vs. 666.0±220.8/mL, p<0.001). Furthermore, both HDL-induced COX-expression and PGI2 release were concentration-dependent, particularly in HDL from T2DM (Figure 2D, E and F).

### Pertussis toxin attenuates HDL-induced COX-2 expression and PGI-2 release

**Figure 3:**
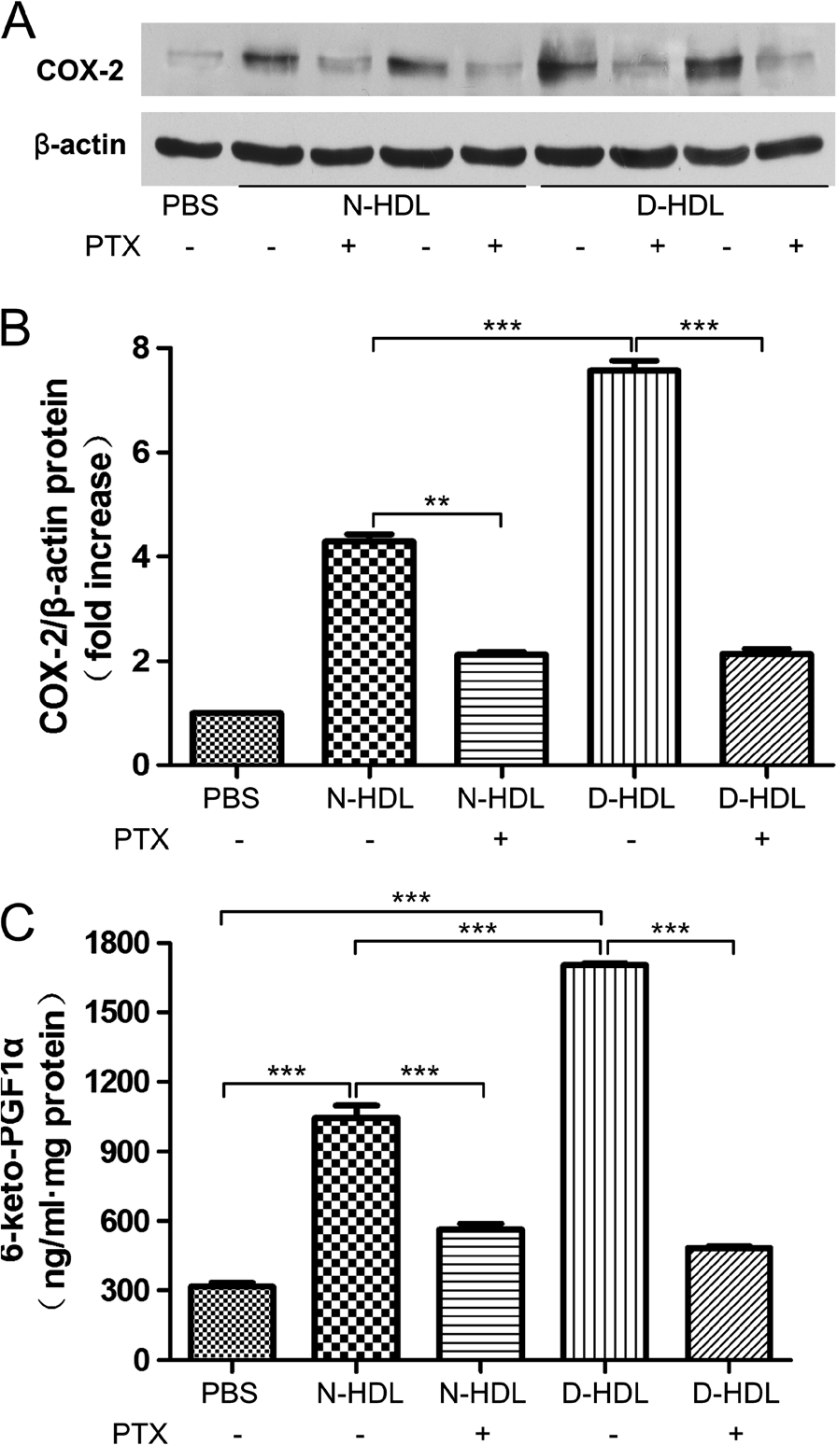
**Pertussis toxin (PTX) attenuates HDL-induced HUVEC COX-2 expression and PGI-2 release.** Cells were preincubated with PTX (2 ng/ml) for 24 hours. Cells were further incubated with 30 μg/ml of N-HDL or D-HDL for 6 hours. The expression of COX-2 was measured by Western blotting (**A**, **B**) and the PGI-2 release was determined by the competitive ELISA (**C**). In all experiments above, PBS was used as vehicle control. Data are expressed as the means ± SEM of three independent experiments. Student’s *t* test. ** p < 0.01, *** p < 0.001.

To investigate whether the contents of S1P carried per HDL particle account for the effects of HDL on the COX-2 expression and PGI-2 release by human vascular endothelial cells, we used pertussis toxin (PTX), a common inhibitor of G-couple protein receptors (G-PCR) to evaluate the blockage. HUVECs were pre-treated with PTX (2 ng/mL) for 24 hours and then incubated with or without HDL isolated from patients with T2DM and control subjects for further 6 hours. The result showed that PTX markedly attenuated HDL-induced COX-2 expression (Figure 3A and B), while the inhibitor also reduced HDL-induced PGI-2 release (Figure 3C, N-HDL+PTX vs. N-HDL, 564.0 ± 43.2 vs. 1047 ± 90.9 pg/mL, p<0.001; D-HDL+PTX vs. D-HDL, 483.8 ± 14.7 vs. 1705 ± 12.3 pg/mL, p<0.001).

### VPC 23109,a special antagonist of S1P receptor (1 and 3) attenuates HDL-induced COX-2 expression and PGI-2 release

**Figure 4:**
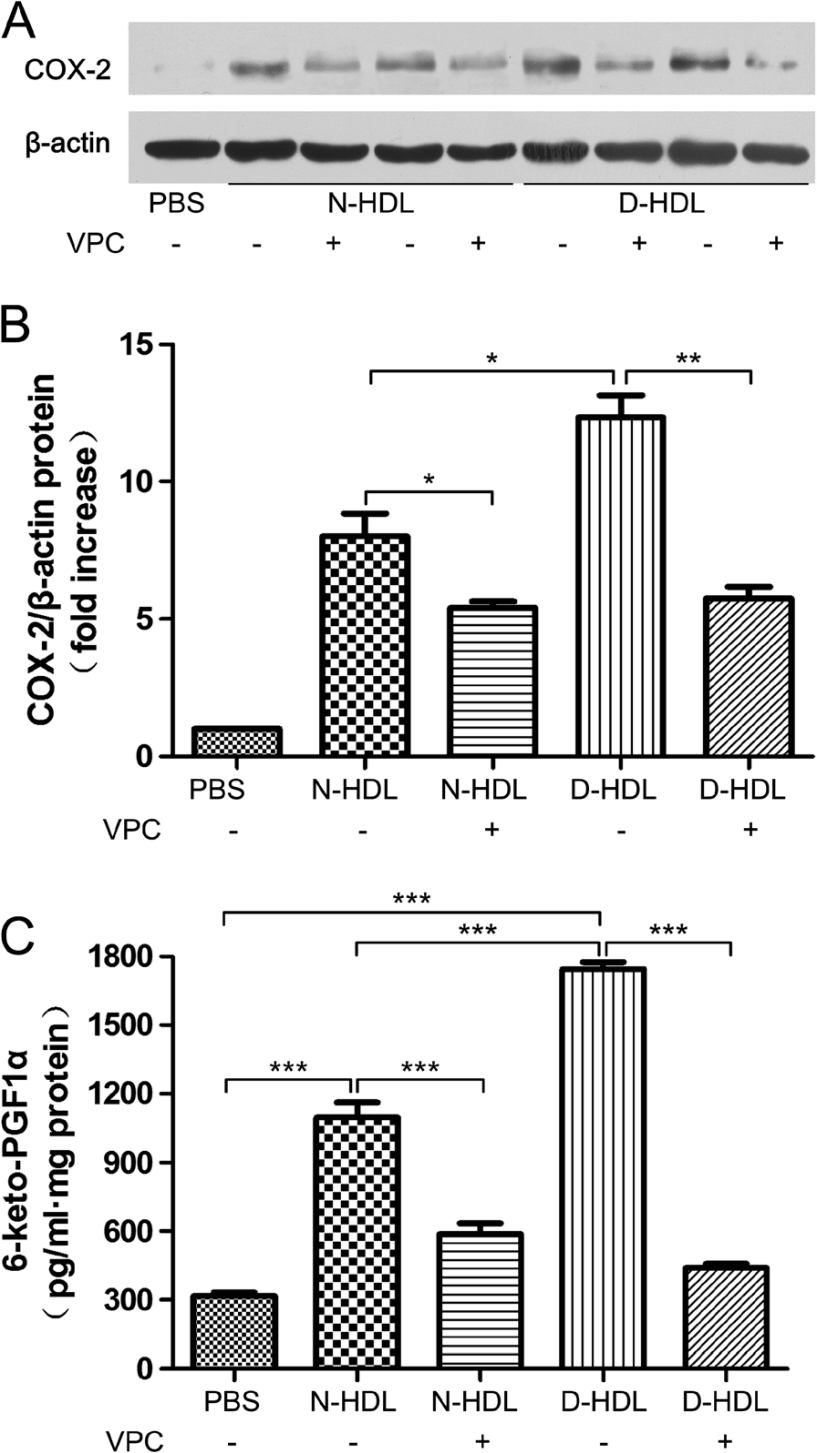
**VPC 23109 attenuates HDL-induced HUVEC COX-2 expression and PGI-2 release.** VPC 23019 attenuated HDL-induced COX-2 expression (**A**, **B**) and PGI-2 release (**C**) by HUVECs. In these experiments, cells were pre-treated VPC 23019 (2 nmol/ml) for 20 minutes and then incubated with 30 μg/ml of N-HDL or D-HDL for further 6 hours. The measurements of COX-2 expression (**A**, **B**) and PGI-2 release (**C**) were same as above. In all experiments above, PBS was used as vehicle control. Data are expressed as the means ± SEM of three independent experiments. Student’s *t* test. * p < 0.05. ** p < 0.01, *** p < 0.001.

S1P induced COX-2 expression by HUVECs, either alone or associated with HDL (Additional file 1: Figure S1). To further investigate the effects of S1P on the HDL-induced responses by human vascular endothelial cells, we used VPC23019 (Avanti Polar Lipids, Alabaster AL), a selective antagonist of S1P receptor 1 (S1PR1) and receptor 3 (S1PR3), which have been shown to be involved in the S1P-mediated processes in HUVECs [22]. HUVECs were pre-treated with VPC23019 (2 nmol) for 20 minutes,then incubated with or without HDL isolated from patients with T2DM and control subjects for further 6 hours. The results showed that VPC23109 diminished the effect of diabetic HDL on COX-2 expression (Figure 4A and B) and PGI-2 release (Figure 4C, N-HDL vs. PBS control, 1096 ± 114.8 vs. 317.6 ± 28.4 pg/mL, p <0.001; D-HDL vs. N-HDL, 1745 ± 50.9 vs. 1096 ± 114.8 pg/mL, p<0.001; N-HDL+VPC vs. N-HDL, 587.1 ± 84.6 vs. 1096 ± 114.8 pg/mL, p<0.001; D-HDL+VPC vs. D-HDL, 440.4 ± 32.0 vs. 1745 ± 50.9 pg/mL, p<0.001).

## Discussion

Previous studies of diabetic HDL have been focusing on its pro-inflammatory role and dysfunctions such as reverse cholesterol transport (3). In diabetes mellitus, disorders of carbohydrate, fat and protein metabolism result in modifications of HDL protein and lipid components, which may lead to functional deficiency of plasma HDL [23]. Consistent high levels of blood glucose may not only cause protein glycosylation, resulting in dysfunction of reverse cholesterol transport and anti-inflammatory effects, but may also cause disorder in lipid metabolism which possibly alters the function of HDL particle in T2DM. These modifications greatly affect clearance of HDL and apoA-I from circulation [24,25]. On the other hand, the increased non-enzymatic glycosylation of the major apolipoprotein, including apoA-I secondary to hyperglycemia impairs cholesterol transport and its anti-inflammatory function [26,27]. In fact, all changes should gradually occur because T2DM is a chronic disease. Therefore, the body of patient tries to compensate the dysfunctional changes at the beginning of abnormal metabolism while disease itself may be a result of decompensation of the body. During the compensated stage, patients usually exhibit few symptoms of diabetes without complications of vascular damage due to the body’s compensatory protective regulations. However, there is insufficient evidence showing whether there are some compensatory protective effects, including the effects of HDL in patients with T2DM. The relationship between the changes of level of HDL-associated S1P and the disease progression, like atherosclerosis, is still unclear due to the lack of longitudinal studies.

In this study, we have generated a body of data supporting the hypothesis that HDL and S1P play some compensatory protective effects in the diabetic status. Subjects of T2DM had higher levels of triglycerides, total cholesterol and LDL-c, but lower levels of HDL-c compared with that of controls. On the other hand, the levels of HDL-associated S1P suggesting that S1P may be one of compensatory mechanisms in the early stage of T2DM. This increased content of S1P associated with HDL is possibly due to alternated capacity of HDL loading more S1P from plasma, or increased concentration of plasma S1P, or both [28]. Also, the levels of S1P associated with HDL from T2DM-As were decreased than early T2DM, suggesting that diabetic HDL may lose its compensatory effects as disease progresses. The levels of S1P associated with diabetic HDL3 were higher than that of native HDL3 implying that this difference of S1P might be because of the alteration of S1P in HDL3. It is now known that diabetes causes alteration in the sphingolipids metabolism [29]. However, these studies have focused on sphingosine, but not S1P, a downstream product of the metabolism. S1P is generated from the phosphorylation of sphingosine catalyzed by two sphingosine kinase isoenzymes, SphK1 and SphK2 [30]. Slight changes in the levels of sphingomyelin, ceramide or sphingosine are able to significantly increase the level of S1P. Studies of S1P biological functions suggest that S1P may play different roles in various conditions. For instance, the increased S1P levels have been observed in the animal models of type 1 diabetes, type 2 ob/ob mice and diabetic humans and implicated in pro-thrombotic and pro-inflammatory effects [24,31]. S1P accounts for several antiatherogenic and anti-inflammatory effects of HDL. S1P also antagonizes endothelial dysfunction by preventing monocyte/endothelial interactions through activation S1PR1 in the type 1 NOD mouse model and has vascular protective properties, whereas S1PR1 activation promotes eNOS activation and nitric oxide production [32]. Our data also showed that HDL with higher content of S1P upregulated COX-2 expression and PGI-2 release by human vascular endothelial cells, two important athero-protective factors [33].

S1P receptors belong to G-coupled receptors while human vascular endothelial mainly express two of S1P receptors, S1PR1 and S1PR3. To investigate whether the effects of HDL on HUVEC were mediated through S1P, we employed a common G-PRC inhibitor pertussis toxin and S1PR1 and 3 specific antagonist VPC 23019. It has been shown in a previous study in which the expression of S1PR1 was higher in diabetes than controls, suggesting that S1P and its receptors might play important role in diabetes [34]. Our data showed that up to 80% HDL-mediated effects on COX-2 expression and PGI-2 release was blockaded by these two inhibitors, suggesting that the effects of HDL on HUVEC largely contribute the contents of S1P in HDL. G-PCR is large group of receptors which located on cell membrane. We used PTX as a general blocker of G-PCR. On the other hand, we used VPC23109, a selective blocker of S1PR1 and 3, to narrow down the range of these blockages inhibiting the specific effects of S1P and its receptors. Selective blockage of S1PR1 and 3 seemed weaker than that of general G-PCR inhibitor PTX, particularly in COX-2 expression. A potential explanation is that other G-PCRs except for S1PR1 and 3 may also have similar effects. Previous studies have shown that S1P was associated with apolipoprotein M (apoM) in HDL exerted its biological function [35]. But the effects of S1P associated with apoM in metabolic disease are still unknown. Whether any changes of modification and function of apoM in T2DM needs further investigations.

Normally, with developing of disease, the metabolic process starts dynamically from balance to unbalance,from compensation to decompensation. Similar status may occur in S1P in HDL, while HDL might finally lose the compensatory effects. Some clinical trials have shown that “glycemic lowering therapies” and “intensive lipid lowering treatment” might not decrease mortality caused by macrovascular events in T2DM [36]. Therefore, it is necessary to further understand the potential mechanism of the lipid metabolism in HDL in T2DM, particularly in the early stage of the disease.

We also evaluated other effects of diabetic HDL, especially leucocyte-endothelial interaction. Compared with N-HDL, diabetic HDL enhanced more THP-1 cells to adhere to HUVECs, which is possibly associated with increased expression of adhesion molecules, including ICAM-1 and VCAM-1 (Additional file 1: Figure S2). Previous studies have shown that advanced glycation end products (AGEs) play an important role in impairing endothelial progenitor cell (EPC) functions, which may contribute to the development of vascular diseases in diabetes [37].

In conclusion, our data suggests that diabetic HDL with higher content S1P may exert some protective effects on vascular endothelial system, as a kind of the body’s compensatory mechanism preventing or delaying complications from the disease. Future studies should extend its other protective effects and focus on therapeutic potential in this area.

## Abbreviations

T2DM: Type2 diabetes mellitus; HDL: High-density lipoprotein; S1P: Sphingosine-1-phosphate; COX-2: Cyclooxygenase-2; PGI-2: Prostacyclin I-2; HUVECs: Human umbilical vein endothelial cells; S1PR1: S1P receptor 1; S1PR3: S1P receptor 3.

## Competing interest

The authors declare they have no competing interests.

## Authors’ contribution

As to the contribution of each author, Xunliang Tong, Lemin Zheng and Yining Huang designed the study, analyzed and interpreted the results, and drafted the manuscript; Hui Peng and Jianying Hu were involved in the UPLC-MS/MS performance; Donghui Liu, Liang Ji and Chenguang Niu made substantial contributions to performing the experimental protocol; Jun Ren and Bing Pan were involved in revising the manuscript critically for important intellectual content. All authors participated in the discussion and interpretation of the results and in the final approval of the manuscript submitted.

## Supplementary Material

Additional file 1: Figure S1 S1P associated with BSA and N-HDL enhanced COX-2 expression by HUVECs,which was attenuated by S1PR1 and S1PR3 antagonist, VPC 23019. A and B: HUVECs were pre-treated with antagonist VPC 23019 (2 nmol/ml) for 20 minutes and then incubated with 0.5% BSA, S1P (2 μM of S1P carried by 0.5% BSA), N-HDL (30 μg/ml) and S1P-N-HDL (2 μM of S1P carried by 30 μg/ml of N-HDL) for 6 h to measure the expression of COX-2 by Western blotting. S1P-BSA and S1P-N-HDL significantly increased the expression of COX-2 compared with BSA and N-HDL alone. Figure S2: Diabetic HDL enhanced adhesion molecule expression compared with N-HDL. A and B: HUVECs were incubated with PBS, N-HDL or D-HDL (1 mg/ml) for 6 hours. THP-1 monocytic cells were then overlaid on the cells. Fifteen minutes later, non-adherent THP-1 cells were removed by washing. A: representative photographs showing adhered THP-1 cells at various conditions. B: the numbers of adhered THP-1 cells to pre-treated HUVECs. The results are the means ± SEM of three wells from three independent experiments. C and D: HUVECs were similarly incubated for 6 h with the indicated concentrations of N-HDL or D-HDL for the measurement of ICAM-1 (C) and VCAM-1 (D) protein expression. The results are presented as the mean of SEM of OD volume of three individual experiments. Click here for file

## References

[B1] Neeli H Gadi R Rader DJ Managing diabetic dyslipidemia: beyond statin therapy Curr Diab Rep 2009 9 1 11 17 10.1007/s11892-009-0004-y 19192419

[B2] Plutzky J Viberti G Haffner S Atherosclerosis in type 2 diabetes mellitus and insulin resistance: mechanistic links and therapeutic targets J Diabetes Complications 2002 16 6 401 415 10.1016/S1056-8727(02)00202-7 12477625

[B3] Sorci-Thomas MG Thomas MJ High density lipoprotein biogenesis, cholesterol efflux, and immune cell function Arterioscler Thromb Vasc Biol 2012 32 11 2561 2565 10.1161/ATVBAHA.112.300135 23077142 PMC3793253

[B4] Patel S Drew BG Nakhla S Duffy SJ Murphy AJ Barter PJ Rye KA Chin-Dusting J Hoang A Sviridov D Reconstituted high-density lipoprotein increases plasma high-density lipoprotein anti-inflammatory properties and cholesterol efflux capacity in patients with type 2 diabetes J Am Coll Cardiol 2009 53 11 962 971 10.1016/j.jacc.2008.12.008 19281927

[B5] Calkin AC Drew BG Ono A Duffy SJ Gordon MV Schoenwaelder SM Sviridov D Cooper ME Kingwell BA Jackson SP Reconstituted high-density lipoprotein attenuates platelet function in individuals with type 2 diabetes mellitus by promoting cholesterol efflux Circulation 2009 120 21 2095 2104 10.1161/CIRCULATIONAHA.109.870709 19901191

[B6] Drexel H Aczel S Marte T Vonbank A Saely CH Factors predicting cardiovascular events in statin-treated diabetic and non-diabetic patients with coronary atherosclerosis Atherosclerosis 2010 208 2 484 489 10.1016/j.atherosclerosis.2009.08.026 19748621

[B7] Sato K Okajima F Role of sphingosine 1-phosphate in anti-atherogenic actions of high-density lipoprotein World J Biol Chem 2010 1 11 327 337 10.4331/wjbc.v1.i11.327 21537467 PMC3083937

[B8] Lucke S Levkau B Endothelial functions of sphingosine-1-phosphate Cell Physiol Biochem 2010 26 1 87 96 10.1159/000315109 20502008

[B9] Argraves KM Gazzolo PJ Groh EM Wilkerson BA Matsuura BS Twal WO Hammad SM Argraves WS High density lipoprotein-associated sphingosine 1-phosphate promotes endothelial barrier function J Biol Chem 2008 283 36 25074 25081 10.1074/jbc.M801214200 18606817 PMC2529014

[B10] Argraves KM Argraves WS HDL serves as a S1P signaling platform mediating a multitude of cardiovascular effects J Lipid Res 2007 48 11 2325 2333 10.1194/jlr.R700011-JLR200 17698855

[B11] Liu D Ji L Wang Y Zheng L Cyclooxygenase-2 Expression, Prostacyclin Production and Endothelial Protection of High-density Lipoprotein Cardiovasc Hematol Disord Drug Targets 2012 12 98 10.2174/1871529X11202020098 23030452

[B12] Martinez-Gonzalez J Badimon L Mechanisms underlying the cardiovascular effects of COX-inhibition: benefits and risks Curr Pharm Des 2007 13 22 2215 2227 10.2174/138161207781368774 17691994

[B13] Kimura T Sato K Malchinkhuu E Tomura H Tamama K Kuwabara A Murakami M Okajima F High-density lipoprotein stimulates endothelial cell migration and survival through sphingosine 1-phosphate and its receptors Arterioscler Thromb Vasc Biol 2003 23 7 1283 1288 10.1161/01.ATV.0000079011.67194.5A 12775579

[B14] Sattler K Levkau B Sphingosine-1-phosphate as a mediator of high-density lipoprotein effects in cardiovascular protection Cardiovasc Res 2009 82 2 201 211 19233866 10.1093/cvr/cvp070

[B15] Rodriguez C Gonzalez-Diez M Badimon L Martinez-Gonzalez J Sphingosine-1-phosphate: A bioactive lipid that confers high-density lipoprotein with vasculoprotection mediated by nitric oxide and prostacyclin Thromb Haemost 2009 101 4 665 673 19350109

[B16] Eren E Yilmaz N Aydin O High Density Lipoprotein and it's Dysfunction Open Biochem J 2012 6 78 93 10.2174/1874091X01206010078 22888373 PMC3414806

[B17] Jaffe EA Nachman RL Becker CG Minick CR Culture of human endothelial cells derived from umbilical veins Identification by morphologic and immunologic criteria. J Clin Invest 1973 52 11 2745 2756 10.1172/JCI107470 PMC302542 4355998

[B18] Burnette WN "Western blotting": electrophoretic transfer of proteins from sodium dodecyl sulfate–polyacrylamide gels to unmodified nitrocellulose and radiographic detection with antibody and radioiodinated protein A Anal Biochem 1981 112 2 195 203 10.1016/0003-2697(81)90281-5 6266278

[B19] Chung BH Wilkinson T Geer JC Segrest JP Preparative and quantitative isolation of plasma lipoproteins: rapid, single discontinuous density gradient ultracentrifugation in a vertical rotor J Lipid Res 1980 21 3 284 291 7381323

[B20] Lee MH Hammad SM Semler AJ Luttrell LM Lopes-Virella MF Klein RL HDL3, but not HDL2, stimulates plasminogen activator inhibitor-1 release from adipocytes: the role of sphingosine-1-phosphate J Lipid Res 2010 51 9 2619 2628 10.1194/jlr.M003988 20522601 PMC2918445

[B21] Kingsley PJ Marnett LJ Analysis of endocannabinoids, their congeners and COX-2 metabolites J Chromatogr B Analyt Technol Biomed Life Sci 2009 877 26 2746 2754 10.1016/j.jchromb.2009.05.024 PMC4903075 19505857

[B22] Saba JD Hla T Point-counterpoint of sphingosine 1-phosphate metabolism Circ Res 2004 94 6 724 734 10.1161/01.RES.0000122383.60368.24 15059942

[B23] Onat A Hergenc G Low-grade inflammation, and dysfunction of high-density lipoprotein and its apolipoproteins as a major driver of cardiometabolic risk Metabolism 2011 60 4 499 512 10.1016/j.metabol.2010.04.018 20580781

[B24] Fox TE Bewley MC Unrath KA Pedersen MM Anderson RE Jung DY Jefferson LS Kim JK Bronson SK Flanagan JM Circulating sphingolipid biomarkers in models of type 1 diabetes J Lipid Res 2011 52 3 509 517 10.1194/jlr.M010595 21068007 PMC3035687

[B25] Mooradian AD Dyslipidemia in type 2 diabetes mellitus Nat Clin Pract Endocrinol Metab 2009 5 3 150 159 10.1038/ncpendmet1066 19229235

[B26] Fleisher LN Tall AR Witte LD Miller RW Cannon PJ Stimulation of arterial endothelial cell prostacyclin synthesis by high density lipoproteins J Biol Chem 1982 257 12 6653 6655 7045092

[B27] Liu D Ji L Zhang D Tong X Pan B Liu P Zhang Y Huang Y Su J Willard B Nonenzymatic glycation of high-density lipoprotein impairs its anti-inflammatory effects in innate immunity Diabetes Metab Res Rev 2012 28 2 186 195 10.1002/dmrr.1297 21928330

[B28] Hammad SM Al GM Semler AJ Klein RL Sphingosine 1-phosphate distribution in human plasma: associations with lipid profiles J Lipids 2012 2012 180705 23209911 10.1155/2012/180705 PMC3503336

[B29] Baranowski M Blachnio-Zabielska A Hirnle T Harasiuk D Matlak K Knapp M Zabielski P Gorski J Myocardium of type 2 diabetic and obese patients is characterized by alterations in sphingolipid metabolic enzymes but not by accumulation of ceramide J Lipid Res 2010 51 1 74 80 10.1194/jlr.M900002-JLR200 19617631 PMC2789788

[B30] Hannun YA Obeid LM Principles of bioactive lipid signalling: lessons from sphingolipids Nat Rev Mol Cell Biol 2008 9 2 139 150 10.1038/nrm2329 18216770

[B31] Yeboah J McNamara C Jiang XC Tabas I Herrington DM Burke GL Shea S Association of plasma sphingomyelin levels and incident coronary heart disease events in an adult population: Multi-Ethnic Study of Atherosclerosis Arterioscler Thromb Vasc Biol 2010 30 3 628 633 10.1161/ATVBAHA.109.199281 20032291 PMC2862629

[B32] Whetzel AM Bolick DT Srinivasan S Macdonald TL Morris MA Ley K Hedrick CC Sphingosine-1 phosphate prevents monocyte/endothelial interactions in type 1 diabetic NOD mice through activation of the S1P1 receptor Circ Res 2006 99 7 731 739 10.1161/01.RES.0000244088.33375.52 16960101

[B33] Arehart E Gleim S Kasza Z Fetalvero KM Martin KA Hwa J Prostacyclin, atherothrombosis, and cardiovascular disease Curr Med Chem 2007 14 20 2161 2169 10.2174/092986707781389637 17691954

[B34] Duru EA Fu Y Davies MG Role of S-1-P receptors and human vascular smooth muscle cell migration in diabetes and metabolic syndrome J Surg Res 2012 177 2 e75 e82 10.1016/j.jss.2011.12.012 22480845 PMC3392410

[B35] Christoffersen C Obinata H Kumaraswamy SB Galvani S Ahnstrom J Sevvana M Egerer-Sieber C Muller YA Hla T Nielsen LB Endothelium-protective sphingosine-1-phosphate provided by HDL-associated apolipoprotein M Proc Natl Acad Sci U S A 2011 108 23 9613 9618 10.1073/pnas.1103187108 21606363 PMC3111292

[B36] Koshizaka M Green JB Alexander JH Glycemic management in diabetes and the associated cardiovascular risk: are we helping or hurting our patients? Circ J 2012 76 7 1572 1580 10.1253/circj.CJ-12-0428 22789974

[B37] Li H Zhang X Guan X Cui X Wang Y Chu H Cheng M Advanced glycation end products impair the migration, adhesion and secretion potentials of late endothelial progenitor cells Cardiovasc Diabetol 2012 11 46 10.1186/1475-2840-11-46 22545734 PMC3403843

