# Supplementary material for: High-density lipoprotein of patients with Type 2 Diabetes Mellitus upregulates cyclooxgenase-2 expression and prostacyclin I-2 release in endothelial cells: relationship with HDL-associated sphingosine-1-phosphate
Source: Cardiovasc Diabetol. 2013 Jan 30;12:27. doi: 10.1186/1475-2840-12-27 (PMC3599898; doi:10.1186/1475-2840-12-27)
Supplement: Additional file 1: Figure S1 — S1P associated with BSA and N-HDL enhanced COX-2 expression by HUVECs,which was attenuated by S1PR1 and S1PR3 antagonist, VPC 23019. A and B: HUVECs were pre-treated with antagonist VPC 23019 (2 nmol/ml) for 20 minutes and then incubated with 0.5% BSA, S1P (2 μM of S1P carried by 0.5% BSA), N-HDL (30 μg/ml) and S1P-N-HDL (2 μM of S1P carried by 30 μg/ml of N-HDL) for 6 h to measure the expression of COX-2 by Western blotting. S1P-BSA and S1P-N-HDL significantly increased the expression of COX-2 compared with BSA and N-HDL alone. Figure S2: Diabetic HDL enhanced adhesion molecule expression compared with N-HDL. A and B: HUVECs were incubated with PBS, N-HDL or D-HDL (1 mg/ml) for 6 hours. THP-1 monocytic cells were then overlaid on the cells. Fifteen minutes later, non-adherent THP-1 cells were removed by washing. A: representative photographs showing adhered THP-1 cells at various conditions. B: the numbers of adhered THP-1 cells to pre-treated HUVECs. The results are the means ± SEM of three wells from three independent experiments. C and D: HUVECs were similarly incubated for 6 h with the indicated concentrations of N-HDL or D-HDL for the measurement of ICAM-1 (C) and VCAM-1 (D) protein expression. The results are presented as the mean of SEM of OD volume of three individual experiments. [file 1475-2840-12-27-S1.doc]

**Methods in Data Supplement:**

1. THP-1 cell adhesion assay

THP-1 monocytic cells were washed twice and resuspended in RPMI 1640 containing 0.1% bovine serum albumin (BSA). The cells were overlaid (1.5×10^6^ cells/ml, 500μl/well) on the confluent monolayers of HUVECs that had been grown in 12-well plates and pre-treated with various HDL for 6 hours. After incubation for 15 min at 37°C, non-adherent THP-1 monocytic cells were removed by washing three times with pre-warmed PBS. The numbers of THP-1 monocytic cells adhered to the HUVECs were counted in four places under microscopy at 400×magnification.

2. Determination of cell surface expression of adhesion molecules by Cellular Enzyme Immunoassay

HUVECs were plated on 96-well plates and treated with various HDL for 6 hours. The cells were then washed twice and incubated in ECM media containing 0.1% FBS with test agents for 6 h. Thereafter cells were washed with PBS twice and fixed with PBS containing 3% formamide under 4 °C. The plates were blocked at 4 °C overnight with 5% skim milk powder in PBS. Cell surface expression of adhesion molecules was determined by primary binding with specific rabbit antibody against VCAM-1 or ICAM-1, followed by secondary goat anti-rabbit IgG antibody conjugated with horseradish peroxidase. Quantification was performed by determination of colorimetric conversion at an optical density at 450 nm of 3’,5,5’-tetramethylbenzidine using TMB peroxidase EIA substrate kit (Bio-Rad).

**Figure legends in Data Supplement:**

**Figure 1:** S1P associated with BSA and N-HDL enhanced COX-2 expression by HUVECs，which was attenuated by S1PR1 and S1PR3 antagonist, VPC 23019**.** A and B: HUVECs were pre-treated with antagonist VPC 23019 (2 nmol/ml) for 20 minutes and then incubated with 0.5% BSA, S1P (2 μM of S1P carried by 0.5% BSA), N-HDL (30 μg/ml) and S1P-N-HDL (2 μM of S1P carried by 30 μg/ml of N-HDL) for 6 h to measure the expression of COX-2 by Western blotting. S1P-BSA and S1P-N-HDL significantly increased the expression of COX-2 compared with BSA and N-HDL alone.

**Figure 2:** Diabetic HDL enhanced adhesion molecule expression compared with N-HDL. A and B: HUVECs were incubated with PBS, N-HDL or D-HDL (1 mg/ml) for 6 hours. THP-1 monocytic cells were then overlaid on the cells. Fifteen minutes later, non-adherent THP-1 cells were removed by washing. A: representative photographs showing adhered THP-1 cells at various conditions. B: the numbers of adhered THP-1 cells to pre-treated HUVECs. The results are the means ± SEM of three wells from three independent experiments. C and D: HUVECs were similarly incubated for 6 h with the indicated concentrations of N-HDL or D-HDL for the measurement of ICAM-1 (C) and VCAM-1 (D) protein expression. The results are presented as the mean of SEM of OD volume of three individual experiments.

**Figure1 in Data Supplement:**

**
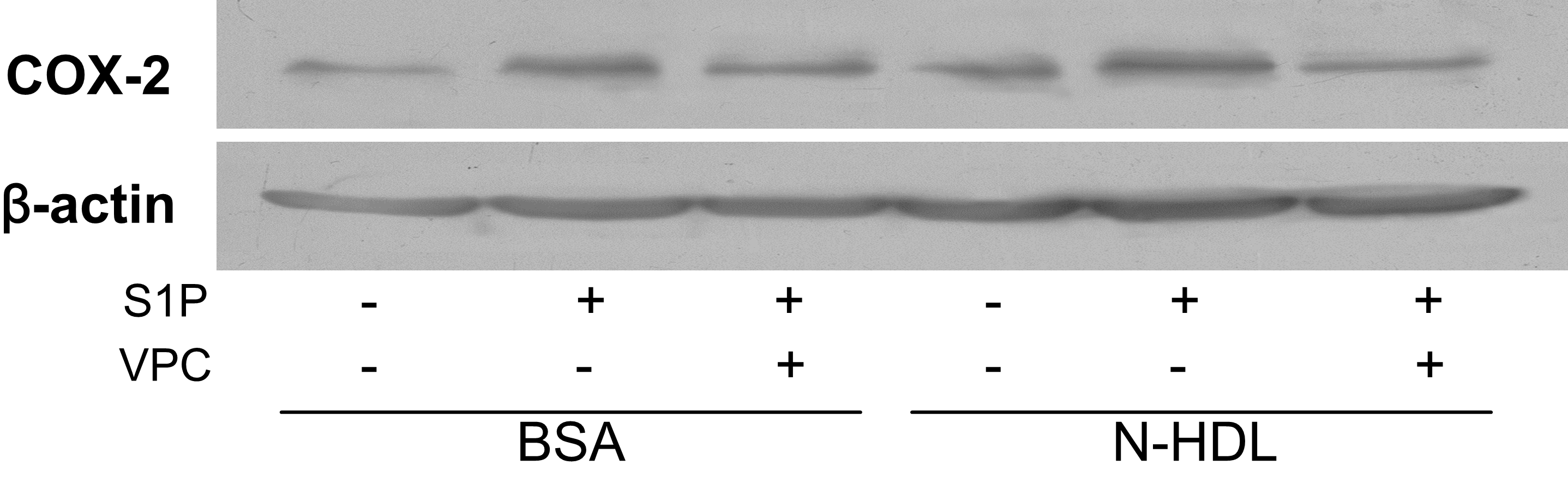
**

**Figure 2 in Data Supplement:**

**
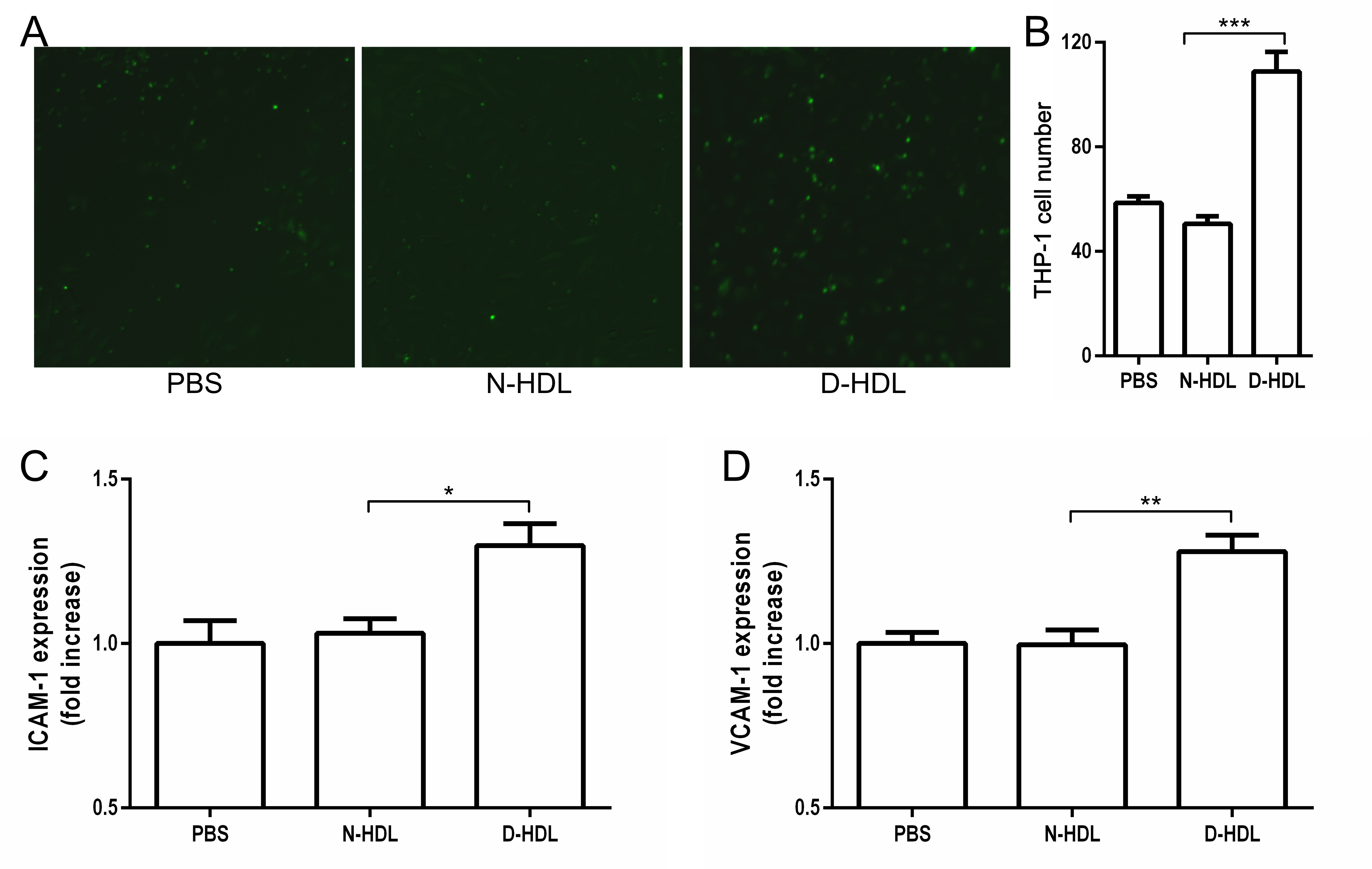
**
